# Approach to Evaluating Reorganization Energies of Interfacial Electrochemical Reactions

**DOI:** 10.1021/acselectrochem.5c00158

**Published:** 2025-07-02

**Authors:** Karnamohit Ranka, Sijia Ke, Chenqi Fan, Jeffrey B. Neaton, Peter Agbo, Frances A. Houle

**Affiliations:** † Liquid Sunlight Alliance, 1666 Lawrence Berkeley National Laboratory, Berkeley, California 94720, United States; ‡ Chemical Sciences Division, 1438 Lawrence Berkeley National Laboratory, Berkeley, California 94720, United States; § Department of Physics, University of California at Berkeley, Berkeley, California 94720, United States; ∥ Department of Chemistry, University of California at Berkeley, Berkeley, California 94720, United States; ⊥ Materials Sciences Division, Lawrence Berkeley National Laboratory, Berkeley, California 94720, United States; # Department of Materials Science and Engineering, University of California at Berkeley, Berkeley, California 94720, United States; ∇ Kavli Energy NanoSciences Institute at Berkeley, Berkeley, California 94720, United States; ○ Molecular Biophysics and Integrated Bioimaging Division, Lawrence Berkeley National Laboratory, Berkeley, California 94720, United States

**Keywords:** interfacial reaction, electrochemistry, reorganization energy, Marcus−Hush−Chidsey, Butler−Volmer, electron-transfer kinetics

## Abstract

Reaction rate coefficients for electron-transfer processes at the electrode–electrolyte interface are commonly estimated by using the Butler–Volmer equation, but their values are inaccurate beyond a few tenths of volts of overpotential. The Marcus–Hush–Chidsey (MHC) formalism yields correct asymptotic behavior of the rate coefficients vs applied overpotential but has complex dependencies on the redox system’s intrinsic parameters, which can be difficult to model or measure. In this work, we bridge the two kinetics formalisms to estimate the reorganization energy, one of the important parameters for the MHC formalism, and investigate its dependence on other intrinsic parameters such as activation barriers, electronic coupling strength, and the density of states of the electrode surface. We examine the sensitivity of the reorganization energy to these parameters, establish some general relationships for accurately predicting rate coefficients using the MHC formalism over a wide range of applied overpotentials, and compare this approach to calculating MHC rate constants with other empirical approaches for the mechanisms of CO_2_ reduction on different metal electrode surfaces.

## Graphical abstract

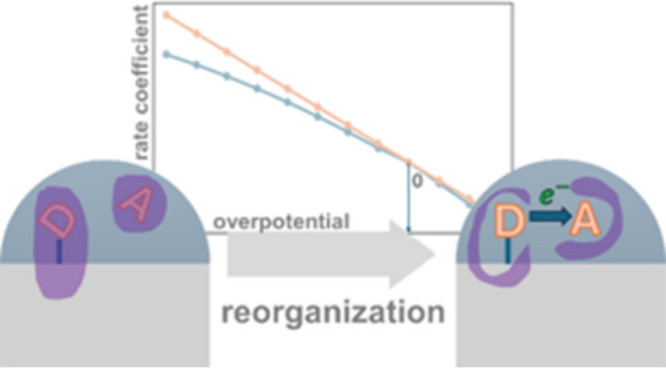

## Introduction

A kinetic description of electrochemical reactions can provide important information about the chemical composition of the systems with time. While such information may be accessible using experimental (spectroscopic) methods, isolating the kinetic effects of some components or reactions over others is often not possible. While Gibbs free energies from theory do provide vital thermodynamic information, they do not elucidate the kinetics of coupled reactions, which depend decisively on the time-dependent composition of the electrochemical system. Modeling the kinetics of such systems can play a crucial role in unraveling reaction pathways and the importance of specific steps in the overall mechanism.^1,2^ However, a kinetics model of any system must reproduce the experimental observations such as the empirical Tafel relationship, which predicts a linear dependence of the overpotential (*η*, defined as the difference between applied and formal potentials, *E – E* ^0^) on the logarithm of the measured electrode current (or current density), *i*.^1,3,4^

In the context of reactions occurring at electrode surfaces, the intrinsic rate coefficient, *k* ^0^, is calculated using transition state theory (TST) and is assumed to govern the equilibrium (*η* = 0) kinetics. The use of TST implies that the thermodynamic end states of electron-transfer within the redox couple are connected by a transition state. This state is an intermediary structure, whose energy and position along the reaction coordinate define a transfer coefficient (also known as a symmetry factor), *α*. The equilibrium current at either the cathode or the anode is called the exchange current, *i*_0_, and its magnitude depends on *k* ^0^ and the equilibrium concentrations of reactants and products of the electrochemical reaction under consideration. The next step in developing the kinetic model is accounting for the experimentally observed dependence of the measured current, *i*, on the applied overpotential, *η*. The kinetics of an *η*-driven heterogeneous electron-transfer process at the electrode surface are commonly modeled using two formalisms: the Butler–Volmer formalism, which employs a phenomenological expression, and the Marcus–Hush–Chidsey formalism, which extends the semi-classical origins of the Marcus electron-transfer theory to reactions at electrode surfaces.^1,5–7^

The Butler–Volmer (BV) equation is an empirical relation between *η* and *i* that uses the Tafel equation to describe cathodic and anodic half-reactions. It holds when mass transport effects in the electrolyte are negligible (this assumption is made in each case discussed in the remainder of the present study).^1,7^ The BV equation reliably predicts rate coefficients near equilibrium conditions (*η* → 0). Owing to its simplicity, the BV expression is routinely applied to various electrochemical mechanisms to predict and model the observed product distributions, surface coverages of electrodes, etc.^8–18^ However, particularly in the heterogeneous case of electron-transfer between a solvated redox couple and a solid electrode surface, it fails to capture even the qualitative behavior of rate coefficients at high overpotentials because of its exponentially increasing dependence on *η* (illustrated in Figure 1).^19–21^ An exponential scaling of the current with *η* requires an infinite supply of electrons from the electrode. This is physically impossible in the limit of high overpotentials due to the limitations imposed by the distribution of the density of electronic states of the electrode surface.^19,22^

**Figure 1:**
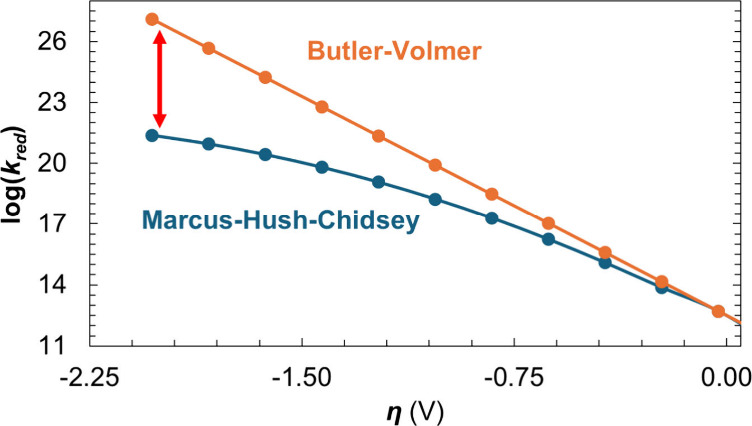
Comparison of rate coefficients for electron-transfer at the Ag cathode (CO_2_* → COOH^**^): Butler–Volmer vs Marcus–Hush–Chidsey. The red arrow emphasizes the discrepancy between rate coefficients calculated using the two formalisms at increasing overpotentials.

Marcus–Hush–Chidsey (MHC) kinetics build upon Marcus theory, considering factors like the energetic cost to rearrange the geometry of the surrounding and participating molecules, known as the reorganization energy, *λ*. It also incorporates the distribution of carriers energetically available for charge-transfer, which may differ slightly in energy from the charge-donor/acceptor state but still have significant probabilities of transfer. One obtains a physical value for the rate coefficient of an electron-transfer reaction only after integrating over this distribution.^23,24^ While this theory accounts for the specific electronic and microscopic properties of the electrode and the redox-couple system, application of MHC kinetics can suffer from errors due to simplifications made (for example, that of a symmetric dependence of the rate coefficient around *η* = 0, analogous to approximating the symmetry factor as 0.5 in the BV formalism).^6^ As shown in Figure 1, the magnitude of the rate coefficient reaches a limiting value as *η* increases, reflecting the finite availability of carriers in the electrode (details in the Supporting Information).

While the two formalisms may seem different, the rate coefficients calculated using the BV and MHC formalisms should converge in the low-overpotential limit, i.e., the two formalisms must agree near and at equilibrium of the electrochemical step.^25^ This agreement presents an opportunity to calculate the complex quantity *λ* using BV parameters and to derive empirical relations to facilitate the application of the more complicated but physically accurate MHC kinetics. In the present study, we provide a way to both estimate *λ* for an adsorbed redox couple and examine the dependence of the estimated *λ* on the accuracy of the quantities often computed from electronic structure calculations. We illustrate with an example of an electrochemical reduction mechanism how this allows for reliable prediction of electrochemical rate coefficients through the application of MHC kinetics, in contrast to the BV equation, over a wide range of overpotentials. We also compare reorganization energies estimated as described in this work from activation barriers and *E* ^0^s to those calculated directly in a separate study.

## Theoretical Framework

### Kinetics of Electron-Transfer Driven by Overpotential

The rate coefficient of an elementary electrochemical reaction involving the transfer of an electron between end states of a redox couple depends on the applied overpotential for both the BV and MHC formalisms. However, in the case of no driving force (zero applied overpotential), the kinetics of the electron-transfer step are dictated by its formal redox potential, *E* ^0^, which determines the intrinsic rate coefficient, *k* ^0^. Let us consider the cathode half-reaction

A1 $$O+e^{-}\to R$$

­(Note: while the reaction above is formally in equilibrium, it lies far to the right for a cathodic half-reaction. Therefore, the rest of the discussion concerning reaction [Disp-formula eqA1] assumes irreversible reduction of *O* to *R*.)

We assume that the electron-transfer between *O* and *R* occurs via formation of a transition state, where the intrinsic rate coefficient is given by the TST at a given temperature *T* as follows:

1 $$k_{TST}^{0}=\left(\frac{k_{B}T}{h}\right)\;\exp\left(-\frac{\Delta G_{a}}{k_{B}T}\right)$$

where *h* is Planck’s constant , *k*_*B*_ is the Boltzmann constant, and Δ*G*_*a*_ is the activation energy. The BV equation asserts that, at an overpotential *η*, the rate coefficient is given as

2 $$k_{red}^{BV}=k_{TST}^{0}\;\exp\left(-\frac{\alpha F\eta n_{e}}{RT}\right)$$

where *n*_*e*_ is the number of electrons transferred in the step, *R* is the ideal gas constant, and *α* is a symmetry factor determining the likelihood of the electron-transfer direction upon formation of the transition state complex. Thus, the rate coefficient of the electron-transfer step increases exponentially with the applied overpotential. (Note: the units of *k*_*red*_^*BV*^ in eq 2 are s^–1^. The units of BV rate coefficients of heterogeneous electrochemical reactions are stated in terms of cm/s, for which the expression in eq 2 can be multiplied by the thickness of the electrode–electrolyte interface at which the redox reaction under consideration is occurring.)

The Marcus–Hush theory, on the other hand, considers the rate of electron-transfer between the electrode and the *O*/*R* redox couple to be dependent on the reorganization of the solvent surrounding *O* and *R*, termed the outer sphere, as well as the reorganization of their immediate surroundings (e.g. ligands), termed the inner sphere.^5,26^ This argument assumes negligible reorganization of the electron-transfer moieties themselves. That is, there is minimal overlap of the participating orbitals on the electron donor and acceptor for the purpose of the electron-transfer. For electron-transfer reactions occurring at the electrode surface, it has been suggested that the rate coefficient of transfer depends on the electronic structure of the electrode and that the rate coefficient of non-diffusion-controlled electron-transfer saturates with increasing overpotential.^19,22^ This leads to the Marcus–Hush–Chidsey (MHC) formalism, wherein the electron-transfer rate coefficient at the electrode–electrolyte interface depends on the electrode surface density of states (*ρ*), the reorganization energy of inner and outer spheres of the redox couple (*λ* = *λ*_*i*_ + *λ*_*o*_), and the electronic coupling strength between the end states of the redox couple (*H*_*OR*_):^6^

3 $$k_{MHC}^{0}=\frac{2\pi^{3/2}}{h}\rho {H_{OR}}^{2}\sqrt{\frac{k_{B}T}{\lambda }}\;\exp\left(-\beta \left(r_{a}-r_{0}\right)\right)\;\exp\left(-\frac{\lambda }{4k_{B}T}\right)$$

4 $$k_{red}^{MHC}=k_{MHC}^{0}I\left(\eta ,\lambda \right)$$

*I*(*η*, *λ*) is an integral that accounts for the distribution of electronic states of the electrode metal surface:

$$I\left(\eta ,\lambda \right)=\int_{-\infty }^{+\infty }\exp\left(-\frac{{\left(x-\lambda -\eta n_{e}\right)}^{2}}{4\lambda k_{B}T}\right)\frac{1}{1+\exp\left(x/k_{B}T\right)}\;dx$$

The quantities *r*_*a*_ and *r*_0_ represent the distance and the distance of closest approach between the redox couple and the electrode surface, respectively. *β* represents the electronic coupling attenuation coefficient, and *ρ* is the averaged density of electronic states of the cathode surface.^27^ It is important to note that using an average *ρ* is an approximation and can lead to erroneous values for *k*_*red*_^*MHC*^ away from the equilibrium (*η* ≫ 0).^24^ The present study considers the *η* → 0 conditions to determine *λ*. Here, we assume that the *O*/*R* redox couple is adsorbed on the metal cathode surface. Thus, the distance between the redox couple and the electrode surface is the same as the distance of the closest approach to the electrode surface, and the exponential term involving the difference between these two distances in eq 3 reduces to 1. Bazant and co-workers^20^ have proposed a simplified formula for the integral *I*(*η*, *λ*):

5 $$I\left(\eta ,\lambda \right)=\frac{\sqrt{\frac{\pi \lambda }{k_{B}T}}}{1+\exp\left(-\frac{\eta n_{e}}{k_{B}T}\right)}\;\mathrm{erfc}\left(\frac{\frac{\lambda }{k_{B}T}-\sqrt{1+\sqrt{\frac{\lambda }{k_{B}T}}+{\left(\frac{\eta n_{e}}{k_{B}T}\right)}^{2}}}{2\sqrt{\frac{\lambda }{k_{B}T}}}\right)$$

Combining eqs 4 and 5 gives the MHC rate coefficient for reaction [Disp-formula eqA1] at a metallic electrode surface (in units of s^–1^):

6 $$k_{red}^{MHC}=k_{MHC}^{0}\left(r_{a}=r_{0}\right)\frac{\sqrt{\frac{\pi \lambda }{k_{B}T}}}{1+\exp\left(-\frac{\eta n_{e}}{k_{B}T}\right)}\;\mathrm{erfc}\left(\frac{\frac{\lambda }{k_{B}T}-\sqrt{1+\sqrt{\frac{\lambda }{k_{B}T}}+{\left(\frac{\eta n_{e}}{k_{B}T}\right)}^{2}}}{2\sqrt{\frac{\lambda }{k_{B}T}}}\right)$$

### Electron-Transfer Reorganization Energy at Zero Overpotential

As eqs 2 and 6 describe the (first order) rate coefficients for the same reaction [Disp-formula eqA1], their equilibrium rate coefficients, or exchange current densities, must be the same:^25^

$$k_{TST}^{0}=k_{red}^{MHC}\left(\eta =0\right)$$

Substituting the full expressions in eqs 2 and 6 and simplifying, we get

7 $$\frac{k_{B}T}{2\pi^{3/2}\rho {H_{OR}}^{2}}\;\exp\left(-\frac{\Delta G_{a}}{k_{B}T}\right)=\sqrt{\frac{k_{B}T}{\lambda }}\;\exp\left(-\frac{\lambda }{4k_{B}T}\right)\times \xi \left(\lambda \right)$$

where

8 $$\xi \left(\lambda \right)=\sqrt{\frac{\pi \lambda }{k_{B}T}}\;\mathrm{erfc}\left(\frac{\frac{\lambda }{k_{B}T}-\sqrt{1+\sqrt{\frac{\lambda }{k_{B}T}}}}{2\sqrt{\frac{\lambda }{k_{B}T}}}\right)$$

*ξ*(*λ*) is typically very small compared to *k*_*MHC*_^0^ (eq 3): the highest value of *ξ*(*λ*)/*k*_*MHC*_^0^ is of the order of 10^–18^ within the range of parameters considered. Equation 7 can be used to determine the value of the total reorganization energy, *λ*, if the intrinsic quantities like the activation barrier, electronic coupling strength, and density of states (DOS) are known. We minimize the residual value of eq 7 by varying *λ*. The nonzero value of *λ* that minimizes the residual below the specified threshold is referred to as the optimized *λ*. Given the dependence on other parameters in eq 7, we examine the extent to which these quantities can influence the value of the optimized *λ*. The density of states *ρ* is chosen as the averaged first-principles calculated density of states between −10 and 4 eV relative to the Fermi level of the electrode metal surface. The computational details can be found in the Supporting Information (Supplementary Note SN1).

## Results and Discussion

### Reduction of Adsorbates on Metallic Electrodes: Relationships and Trends

Activation barriers are crucial to accurately determining the equilibrium rate coefficients and, thus, the kinetics of interfacial electrochemical reactions accurately. These can be determined through atomistic modeling or variable temperature experimental measurements.^10,28–32^ Such numbers are not reliably known for elementary reaction steps owing to various differences in laboratory as well as simulation approaches even among studies of a specific reaction, such as CO_2_ reduction on a Cu electrode.^10,30,33,34^ Thus, if we are to use eq 7 to determine reorganization energy, it is important to characterize the sensitivity of *λ* to changes in Δ*G*_*a*_, as well as other parameters in eq 7: *ρ*, *H*_*OR*_, and *T*.

We use *ρ* ≈ 7.552 × 10^15^ eV^–1^ for a Cu electrode, assuming an interfacial thickness of 1 nm electrode–electrolyte and an electrode surface area of 1 cm^2^ (Supplementary Note SN1). The redox couple consists of a surface-attached complex and the Cu(111) surface, undergoing a one-electron reduction forming a product that desorbs into the electrolyte. Δ*G*_*a*_ is varied from 0 to 2 eV, reflecting the typical range of activation barriers expected in a realistic interfacial electrochemical reduction mechanism, while the range of values used for *H*_*OR*_ is based on the range of values reported for CO_2_RR adsorbates on a Cu surface: 10^–6^ to 10^–3^ eV.^30^ The calculation parameters and results are presented in Table 1 and Figures 2–4.

**Table 1:** Reorganization Energy Trends for Adsorbate Reduction Reactions on Cu(111) Surface, Calculated Using Eq 7

(i)	λ = *m*Δ*G*_*a*_ + *b*	*H*_*OR*_ = 10^–3^ eV		*H*_*OR*_ = 10^–6^ eV	
		*m*	*b*	*m*	*b*
	*T* = 298 K	1.9969	1.4479	1.9945	0.7428
	*T* = 350 K	1.9963	1.6912	-	-
	*T* = 1000 K	1.9926	4.656	-	-
(ii)	*λ* = *nT* + *c*	*H*_*OR*_ = 10^–3^ eV		*H*_*OR*_ = 10^–6^ eV	
		*n*	*c*	*n*	*c*
	Δ*G*_*a*_ = 0.1 eV	0.0046	0.2887	0.0022	0.2867
	Δ*G*_*a*_ = 0.7 eV	0.0046	1.4861	0.0022	1.4825
	Δ*G*_*a*_ = 1.15 eV	0.0046	3.0857	0.0022	3.0954
(iii)	*λ* = *o* ln(*H*_*OR*_) + *d*	*T* = 298 K		*T* = 350 K	
		*o*	*d*	*o*	*d*
	Δ*G*_*a*_ = 0.1 eV	0.102	2.3529	0.1197	2.7188
	Δ*G*_*a*_ = 0.7 eV	0.1024	3.5525	0.1202	3.9185
	Δ*G*_*a*_ = 1.5 eV	0.1026	5.1518	0.1205	5.5177

**Figure 2:**
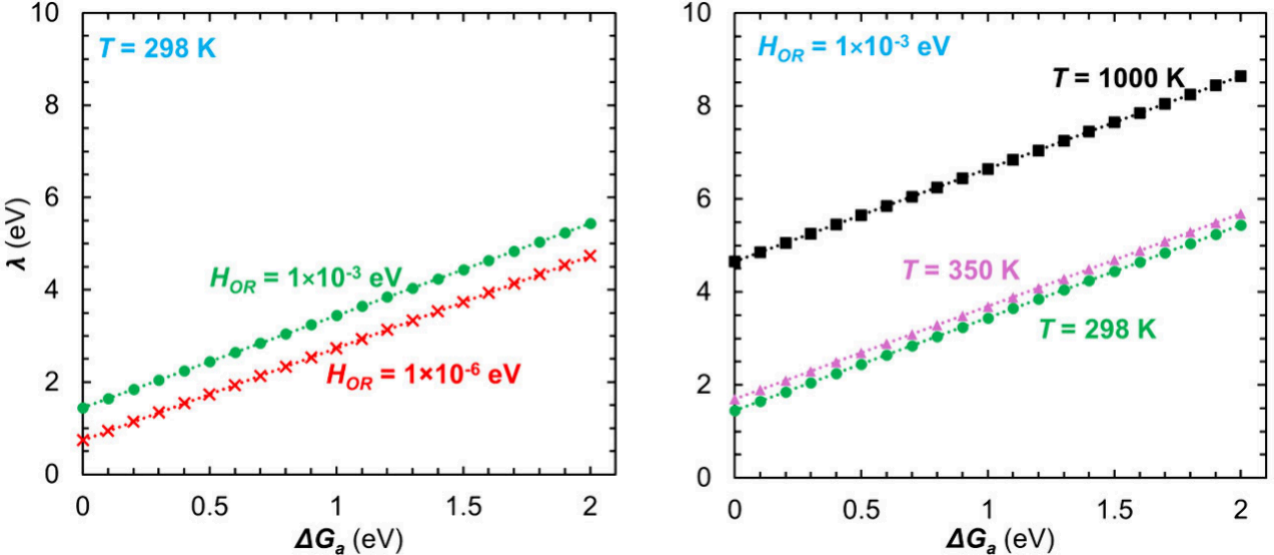
Reorganization energy at Cu electrode surface plotted as a function of free energy of activation for differing electronic coupling strength at *T* = 298 K (left) and temperature at *H*_*OR*_ = 1 × 10^–3^ eV (right). More information on the linear relations can be found in section (i) of Table 1.

Counterintuitively, given the complexity of eq 7, Figure 2 and Table 1 show that *λ* has a linear dependence on Δ*G*_*a*_, with *λ* ≈ 2Δ*G*_*a*_ + *b*. The intercept *b* varies with the values of both *T* and *H*_*OR*_, approximately directly as *T* and inversely as ln­(*H*_*OR*_). Such a dependence indicates that *b* has an important physical meaning and that it emerges as an intrinsic, non-empirical parameter from eq 7. It represents the reorganization needed for the electron-transfer in the absence of an activation barrier and is thus indicative of a minimum reorganization needed for electron-transfer owing to the electronic structure of the electrode and the microenvironment.

As previously mentioned, eq 7 shows that *b*, and thus *λ*, depends on *T*. To examine the extent of this dependence, we plot *λ* values optimized at temperatures ranging from 100 to 1150 K. While this temperature range is not relevant for electrochemical systems, it is examined here as a way to assess the sensitivity of the optimized *λ* toward the system temperature. Figure 3 and Table 1 show a linear dependence of *λ* on *T*: *λ* ≈ *nT* + *c* with *n* ≈ 0.005 eV for *H*_*OR*_ = 10^–3^ eV and *n* ≈ 0.000 eV for *H*_*OR*_ = 10^–6^ eV. The dependence on Δ*G*_*a*_ is incorporated into the intercept *c*, which shows a strong dependence on variation in Δ*G*_*a*_. In fact, the change in *c* corresponds to roughly twice the change in Δ*G*_*a*_. The dependence of *λ* on *T*, as reflected in the slope values, is not as strong as that on the free energy of activation. Furthermore, as the slope of *λ*(*T*) is dependent on the electronic coupling strength, the dependence of *λ* on *H*_*OR*_ is examined in Figure 4 and Table 1. The plots reflect a linear dependence of *λ* on ln­(*H*_*OR*_): *λ* ≈ 0.1 ln­(*H*_*OR*_) + *d*. Here, too, the changes in intercept *d* are roughly twice the changes in Δ*G*_*a*_. The slopes of *λ*(ln­(*H*_*OR*_)) and *λ*(*T*) show that the dependencies of *λ* on *T* and *λ*(ln­(*H*_*OR*_)) are weaker than that on Δ*G*_*a*_, implying that the changes in the value of the determined Δ*G*_*a*_ affect the changes in the value of optimized *λ* in eq 7 the most.

**Figure 3:**
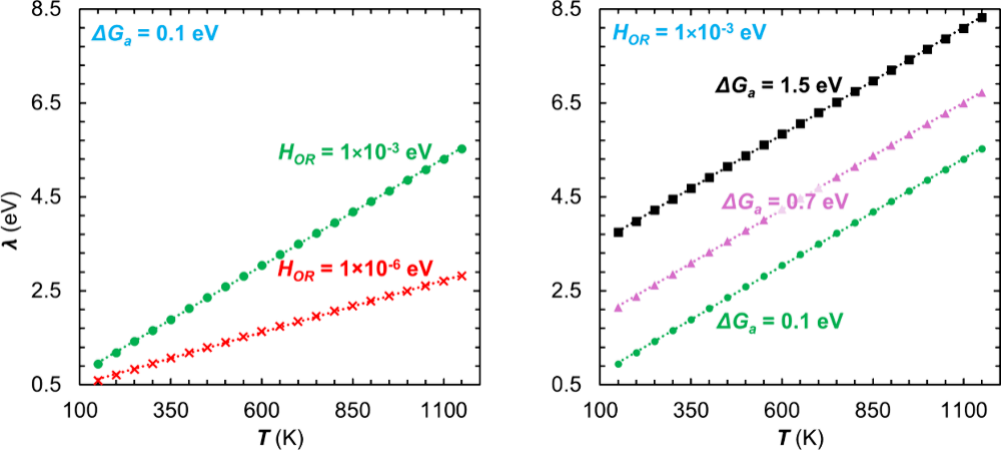
Reorganization energy plotted as a function of temperature for differing electronic coupling strength for Δ*G*_*a*_ = 0.1 eV (left) and Gibbs free energy of activation for *H*_*OR*_ = 1 × 10^–3^ eV (right). More information about the linear relations can be found in section (ii) of Table 1.

**Figure 4:**
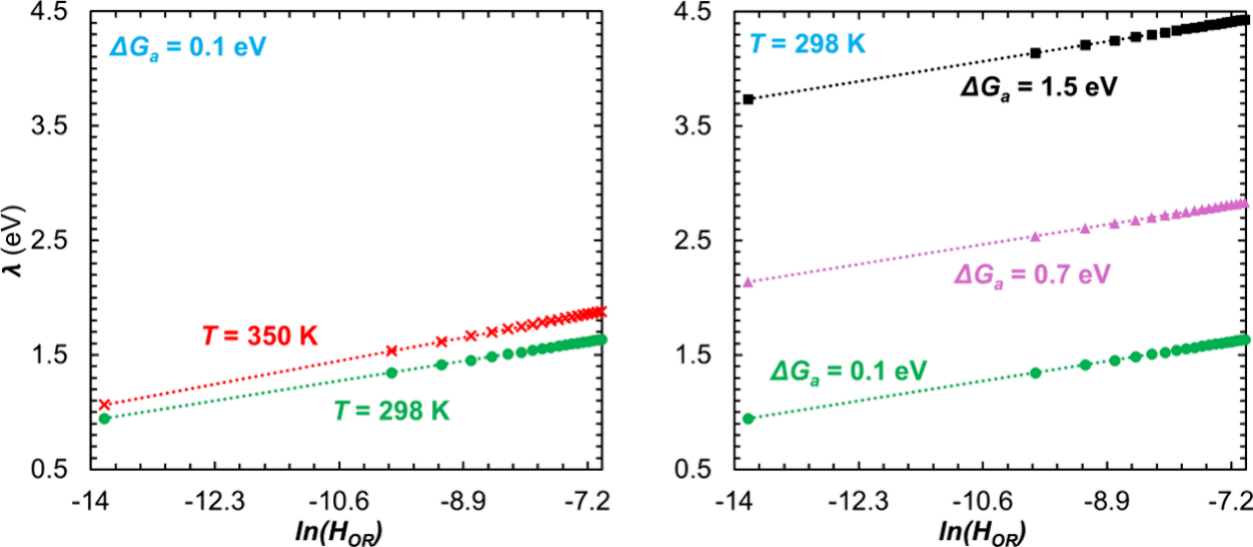
Relationships between reorganization energy and the electronic coupling strength for differing temperature for Δ*G*_*a*_ = 0.1 eV (left) and Gibbs free energy of activation for *T* = 298 K (right). More information about the linear relations can be found in section (iii) of Table 1.

The dependence of *λ* on the average density of states, *ρ*, between two different metals was not observed to vary significantly: the changes were observed to be of the order of 10^–2^ eV in the reorganization energies computed for Ag (Table S1) when compared to those computed for Cu with similar values of other parameters. We found that for the relationship between *λ* and Δ*G*_*a*_, Δ*H*_*OR*_ does not affect the intercept *b* significantly: the maximum difference in the intercept of the linear relationship between *λ* and Δ*G*_*a*_ is Δ*b*(Cu, Ag) ≈ 0.07 eV for *T* = 1000 K and *H*_*OR*_ = 1 × 10^–3^ eV; for the relationship between *λ* and *H*_*OR*_, Δ*d*(Cu, Ag) ≈ 0.02 eV for *T* = 298 K and Δ*G*_*a*_ ranging from 0.1 to 1.5 eV. The small changes in the values of the intercepts *b* and *d* are due to the small relative difference in *ρ* between Cu and Ag metals; this effect is expected to compound for larger relative variation in values of *ρ* (e.g., for materials with significantly different electronic structures).

While the linear relationships thus obtained are useful in estimating the reorganization energy against variations of single parameters at a time, in practice, more than one parameter can simultaneously vary with changing electrode material and adsorbates. Accordingly, the data in Table 1 indicate that the intercepts are often strongly dependent on the other parameters that are fixed when calculating the linear relations. Therefore, we plot in Figure 5 *λ* as a function of Δ*G*_*a*_ and *H*_*OR*_, the two most influential parameters for determining *λ* as outlined above, over four orders of magnitude of the electronic coupling strength, for different values of *T*. This is the range used in the present study for the specific case of a Cu electrode to help deduce general trends of variation in the reorganization energy. The data show that as the Gibbs free energy of activation for an electron-transfer step rises and as the potential free energy surfaces of the initial and final states of a redox couple are coupled more strongly, the energy spent in reorganizing the molecular geometries to facilitate electron-transfer at the electrode interface increases.

**Figure 5:**
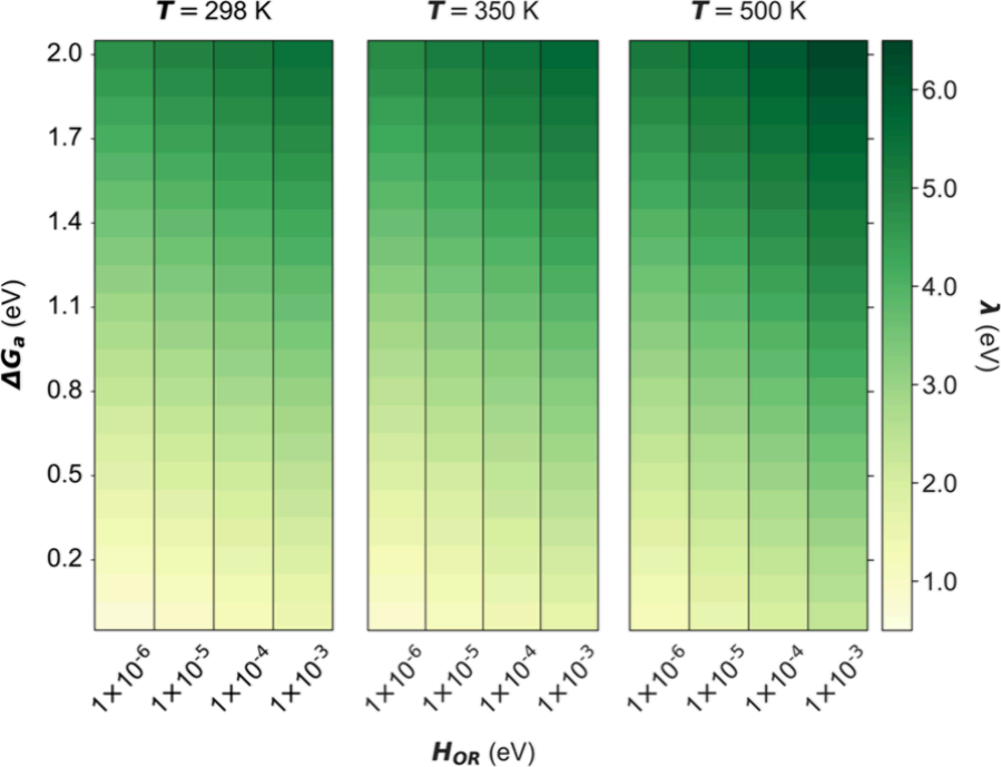
Heatmap showing dependence of the optimized reorganization energy on both the Gibbs free energy of activation and the electronic coupling strength, for reported orders of magnitude^30^ of the electronic coupling strength for an adsorbate on a Cu electrode surface at temperatures of 298 K (left), 350 K (middle), and 500 K (right).

The relationships extracted out of the more complex expression in eq 7 give information about the bounds for the reorganization energy when the bounds on other parameters are known. They also reveal surprisingly near-perfect linear dependencies of reorganization energy on the parameters considered. Equation 7 presents an opportunity to employ Marcus–Hush–Chidsey kinetics when the activation free energy barriers are known for the system of reactions under consideration. It also provides a way to test whether the parameters measured are internally consistent when the reorganization energies and either the activation barriers or the electronic coupling strengths are known. A discrepancy may imply that electron-transfer might not be the rate-controlling factor in the kinetics of the system under study. More generally, the analysis presented here facilitates the application of Marcus–Hush–Chidsey kinetics to various electrochemical reactions on different, well-characterized electrode surfaces. The values of optimized reorganization energies reveal the high energetic penalty incurred upon transferring electrons across metal electrode–aqueous electrolyte interfaces. This is supported by electronic structure calculations of interfacial reorganization energies reported previously.^33,34^ The relationships reported here for a Cu electrode surface were compared with those calculated for a Ag electrode surface, and the changes in trends were insignificant. Thus, the relationships reported here are expected to hold for other electrochemical systems with surface electronic DOS and adsorbate/surface redox couple electronic coupling strengths of similar magnitudes.

The simple linear relationships shown in Figures 2–4 are remarkable given the algebraic complexity of eq 7. The origins of these trends and scaling of slopes and intercepts with key variables are worthy of further analysis, which may provide a deeper understanding of the connections among experimentally observed electrochemical quantities, electronic structure, and key chemical quantities such as reorganization energies.

### CO_2_ Reduction on a Ag Cathode: Comparison of Rate Coefficients

To examine and directly compare different formalisms for analyzing electrochemical kinetics with the present approach, we consider a simple reaction scheme of interfacial electron-transfer on a metallic electrode. It involves a two-electron reduction of CO_2_ to CO combined with hydrogen evolution on a Ag cathode. The corresponding electrochemical reaction scheme is as follows, where we start with adsorbed CO_2_, CO_2_*, on a Ag(111) surface:^9,35^

$${{\mathrm{CO}}_{2}}^{*}+\;*\;+H_{2}O+e^{-}\to {\mathrm{COOH}}^{**}+{\mathrm{OH}}^{-}$$ B1

B2 $${\mathrm{COOH}}^{**}+e^{-}\to \mathrm{CO}+{\mathrm{OH}}^{-}+2\;*$$

B3 $$*\;+H_{2}O+e^{-}\to H^{*}+{\mathrm{OH}}^{-}$$

B4 $$H^{*}+H_{2}O+e^{-}\to H_{2}+{\mathrm{OH}}^{-}+\;*$$

In this scheme, ∗ denotes an available catalytic site on the Ag surface. Adsorbed species are denoted with superscripts * and ** (indicating species singly adsorbed on one catalytic site and doubly adsorbed on two neighboring catalytic sites, respectively), and all other species in the scheme are assumed to be dissolved in the electrolyte, except for *e* ^–^, which is assumed to be embedded in the electrode surface. We use experimentally measured MHC parameters for CO_2_ reduction on polycrystalline Au by Zhang and co-workers,^21^ a reasonable approximation given the similarity of the mechanism on the two metals.^36^

The parameters used to calculate MHC rate coefficients for scheme B are listed in Table 2. We evaluate four different rate coefficients for each of the four steps in three ways: the Butler–Volmer formalism (*k*_*red*_^*BV*^), the Marcus–Hush–Chidsey formalism described above (*k*_*red*_^*MHC*^), and a Marcus–Hush–Chidsey formalism with prefactor parameters and expression from the Au study by Zhang and co-workers^21^ (*k*_*red*_^*Z*20*i*^), which employs a rate coefficient determined at high overpotential, *k*_∞_, extracted from experimental data. The expression from Zhang et al. used for calculating *k*_*red*_^*Z*20*i*^ is

9 $$k_{red}^{Z20i}=k_{\infty }\left(T\right)\sqrt{\frac{RT}{4\pi \lambda_{i}}}\times I\left(\eta ,\lambda \right)$$

where *i* in the superscript of *k*_*red*_^*Z*20*i*^ and the subscript of *λ*_*i*_ represents how the reorganization energy is sourced and *I*(*η*, *λ*) is given by eq 5. Using *k*_∞_ from Zhang et al., we could not find any *λ*_*opt*_ values, calculated using the procedure outlined in the section Electron-Transfer Reorganization Energy at Zero Overpotential and the *η*-independent prefactor in eq 9, for which eq 7 holds. Given this finding, we instead consider two possible choices for reorganization energy for the purpose of calculating rate coefficients according to eq 9: (1) a generic value for inner sphere electron-transfer reorganization energy in a homogeneous medium, *λ*_*gen*_,^37^ to calculate *k*_*red*_^*Z*20*gen*^, and (2) *λ*_*opt*_ calculated using *k*_*MHC*_^0^ instead of *k*_∞_, to calculate *k*_*red*_^*Z*20*opt*^. Data measured with an electrolyte composition of 0.1 M NaHCO_3_ + 0.5 M NaClO_4_ in Zhang et al. are used: *k*_∞_ = 1.42 × 10^–3^ cm/s, for an electrode of surface area 1.53 cm^2^ and an assumed interfacial thickness of 1 nm. For comparison with *k*_*rad*_^*MHC*^, *k*_∞_ is divided by the interfacial thickness of 1 nm to yield *k*_∞_ in units of s^–1^: *k*_∞_^′^ = 1.42 × 10^4^ s^–1^.

**Table 2:** Parameters Used to Calculate Rate Coefficients for Scheme B on the Ag(111) Surface

Parameter	Value
[H_2_O]_*int*_	5.25 M^a^
[∗]	1.66 M^b^
*E*	–1.4 V vs SHE
*ρ*	5.132 × 10^15^ eV^–1^ ^c^
*H*_*OR*_	5.6 × 10^–4^ eV^d^
*λ*_*gen*_	1 eV

a Assumes 4 waters immediately adjacent to a surface metal atom. Only these waters can dissociate to transfer a proton or H atom to the surface. They are instantaneously replaced after they are consumed (steady-state).

b Approximates a surface coverage of 10^14^ cm^–2^.

c *ρ* is calculated for Ag(110) surface but is assumed to be close in magnitude to that of Ag(111) surface (Supplementary Note SN2).

d *H*_*OR*_ for Ag is computed assuming that the electronic coupling strength is proportional to the binding energy of CO on Ag(111) surface, −0.28 eV, compared to that on Cu(111) surface, −0.5 eV, for which *H*_*OR*_ is assumed to be 1 × 10^–3^ eV.^38^

In Table 3, we list the rate coefficients calculated for steps [Disp-formula eqB1]–[Disp-formula eqB4] at an applied potential of −1.4 V vs SHE. As the electrolyte is aqueous, H_2_O is considered to not change in concentration during the reaction. Its interfacial concentration, [H_2_O]_*int*_, as well as the concentration of active surface sites, [∗], are used to convert rate coefficients to their proper orders using the respective first order rate coefficients, *k*_*I*_^*i*^:

10 $$k_{red}^{B1}=k_{I}^{B1}\frac{{\left[H_{2}O\right]}_{\mathrm{int}}}{{\left[*\right]}^{2}}$$

11 $$k_{red}^{B2}=k_{I}^{B2}\frac{{\left[H_{2}O\right]}_{\mathrm{int}}}{\left[*\right]};\;k_{red}^{B3}=k_{I}^{B3}\frac{{\left[H_{2}O\right]}_{\mathrm{int}}}{\left[*\right]};\;k_{red}^{B4}=k_{I}^{B4}\frac{{\left[H_{2}O\right]}_{\mathrm{int}}}{\left[*\right]}$$

**Table 3:** Rate Coefficients Calculated for Scheme B, with Optimized Reorganization Energies from the Present Study and Overpotential Values for Applied Potential of −1.4 V vs SHE

Step (*k* Units)	*λ*_*opt*_ (eV)	*η* (V)	*k*_*red*_^*BV*^	*k*_*red*_^*MHC*^	*k*_*red*_^*Z*20*gen*^ (*λ* = 1 eV)	*k*_*red*_^*Z*20*opt*^
B1. CO_2_* → COOH** (cc/mol·s)	2.50	–1.43	6.33 × 10^22^	6.53 × 10^19^	2.60 × 10^7^	8.32 × 10^4^
B2. COOH** → CO (s^–1^)	3.33	–1.05	1.74 × 10^11^	6.37 × 10^9^	2.63 × 10^4^	8.37 × 10^–3^
B3. H_2_O → H* (s^–1^)	4.27	0.25	1.31 × 10^–5^	5.98 × 10^–6^	5.97 × 10^1^	7.36 × 10^–11^
B4. H* → H_2_ (s^–1^)	3.79	–0.38	1.28 × 10^3^	4.47 × 10^2^	2.85 × 10^2^	2.68 × 10^–8^

It is evident that the MHC values for *k*_*red*_^*Z*20*gen*^ and *k*_*red*_^*Z*20*opt*^ which are estimated using measurements at very high overpotential broadly disagree with those estimated using eqs 7 and 8 except for one case. The MHC rate coefficient *k*_*red*_^*Z*20*gen*^ listed for step [Disp-formula eqB3] is higher in value than the corresponding BV rate coefficient, *k*_*red*_^*BV*^, which always constitutes an upper limit under the assumption of absence of mass-transfer effects. The MHC rate coefficients *k*_*red*_^*Z*20*opt*^ are lower than the corresponding BV values; however, in the case of steps [Disp-formula eqB3] and [Disp-formula eqB4], where *η* = 0.25 V and −0.38 V, respectively, the differences between the *k*_*red*_^*Z*20*opt*^ and *k*_*red*_^*BV*^ values are >5 orders of magnitude. The *k*_*red*_^*MHC*^ values for steps [Disp-formula eqB3] and [Disp-formula eqB4] are much closer to the BV rate coefficients, as expected for low overpotentials.

Given the relatively wide range of overpotentials at a single applied potential of −1.4 V vs SHE in Table 3, it is important to examine how the different kinetics formalisms described above behave across a wide range of applied potentials. In Figure 6, we plot the base 10 logarithm of the four different rate coefficients against potential ranging from 0.2 V to −2.0 V vs SHE, providing a broad span of overpotentials at each value of the potential. One can immediately discern the differences in the results from the three ways we have applied MHC kinetics to scheme B. The BV rate coefficient is an upper bound on the MHC rate coefficient for the same elementary electron-transfer step, and the two rate coefficients must be very close to each other near zero overpotential. The MHC formalism introduced in the section Theoretical Framework (eq 6) satisfies both of these conditions. This is not necessarily true for the MHC rate coefficients obtained from eq 9. These differences highlight the numerical inconsistencies that can be introduced in MHC rate coefficient calculations without considering any experiment-specific information. In this case, the disagreement between the numbers raises the question of whether the estimation of *k*_∞_ was affected by factors such as a mass transport limitation to the reduction rate at large overpotentials.

**Figure 6:**
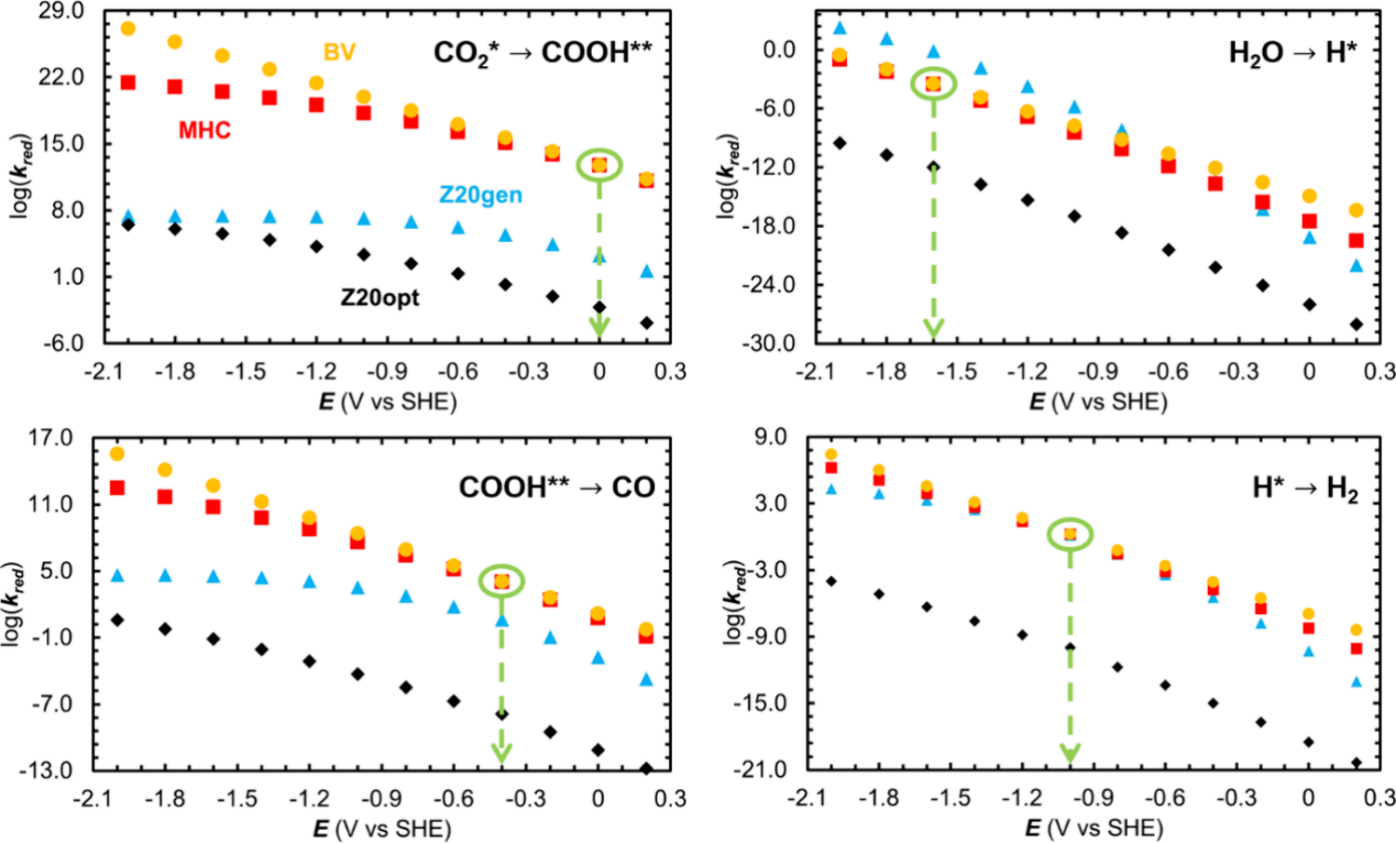
log­(*k*_*red*_) of the reduction half-reaction plotted as a function of the applied potential for steps [Disp-formula eqB1]–[Disp-formula eqB4]. The MHC rate coefficients from the “Z20” study (“Z20gen”, which uses a generic reorganization energy, blue triangles, and “Z20opt”, which uses reorganization energies calculated using this study’s approach, black diamonds) do not always agree with the BV rate coefficient (orange circles) at zero overpotential for a given step, whereas the MHC rate coefficients calculated using the procedure from the present study (red squares) always do so by design. Green ovals and dashed-line arrows point to the formal potential of each step.

### CO_2_ Reduction on a Cu Cathode: Comparison of Reorganization Energies

In this section, we compare reorganization energies calculated using the current study’s approach against a study by Gao and co-workers,^30^ which reports reorganization energies calculated *ab initio*. We use the activation energies calculated using the quadratic relations between the activation barrier and the applied potential in their work, which uses a displaced harmonic oscillator model assuming the same shape of the potential free energy curves for the initial and final states of the reduction reaction considered. We obtain the *E*_0_ values for the elementary steps by estimating the Δ*G*_*rxn*_^0^ values for the Cu(111) surface. We use the reorganization energies determined by Gao et al., *λ*_*R*_, for comparison with the *λ*_*opt*_ values using their activation energies and linear relationships from the current study. We list the values in the Supporting Information (Table S2). There is no simple linear relationship that determines the dependence of *λ*_*R*_ on Δ*G*_*a*_ from the study by Gao et al. (Figure S1, Supporting Information), indicating that there is simultaneous variation in values of other parameters, in particular the electronic coupling strength. The assumption used to obtain the linear relationships in the current study—that the other parameters remain unchanged while only two of them vary (*λ*_*R*_ and Δ*G*_*a*_)—is clearly not valid for this case. However, some of the *λ*_*R*_ values, particularly for the reductions of adsorbed CH* (step C6, 1.98 eV) and CH_2_* (step C7, 1.87 eV), are close to the *λ*_*opt*_ values (1.80 and 1.77 eV, respectively) computed for *H*_*OR*_ = 10^–3^ eV, indicating that for those steps, the electronic coupling strength of 10^–3^ eV is quite close to the *ab initio* electronic coupling strength computed by Gao et al. It is important to note that the present study relies on another assumption that may have drastic effects on calculated *λ*_*opt*_: the use of averaged electronic surface DOS, *ρ*.

### Applicability of the Present Methodology

The calculations presented in Figures 2–5 were made with very specific variations in parameters. We recognize that real systems with heterogeneous surfaces and multicomponent reactive environments are probably not likely to be captured so cleanly. Furthermore, the variation in computed activation energies for similar steps obtained from different sources can add uncertainty and must be considered when analyzing the applicability of kinetics formalisms that depend on their accuracy (Table S3, Supporting Information). Nonetheless, comparisons of idealized trends with those found in realistic systems such as those discussed here emphasize that even simplified relationships are an important tool to evaluate estimated MHC kinetic parameters from both theory and experiments. Improvements to the approach described here would include development of a theoretical interpretation of the values of the intercepts and slopes reported in Table 1 and incorporation of *ρ* in a more rigorous manner to reflect surface heterogeneities. It is also clear that the values of the coupling constants *H*_*OR*_ are crucial to estimating *λ* accurately. It is possible that through implementation of analyses such as the one described here, a good sense of the typical ranges of *H*_*OR*_ and *λ* for various types of electrocatalytic systems will emerge, facilitating our ability to estimate rate coefficients for new systems more easily.

## Conclusion

We propose the combination of a previously known MHC prefactor, a simplified MHC expression introduced by Bazant and co-workers, and the condition that BV and MHC rate coefficients must agree at zero overpotential to obtain an equation that can be used to solve for the reorganization energy of electron-transfer between the adsorbate/electrode surface redox couple. The primary assumptions employed in the present approach are as follows: (1) the exchange current density is limited by the electron-transfer step (no mass transport limitations). (2) The exchange current density for an electron-transfer step is given by Marcus theory (MHC prefactor uses Marcus theory expression). (3) The DOS is independent of electron energies (use of average DOS) and (4) the electronic coupling strength between the end states of the redox couple is independent of electron energies.

We examine simplified linear relationships from the complex equation obtained and observe that the inner sphere reorganization energies thus computed for interfacial reduction reactions are in agreement with previously reported reorganization energies. We also apply this approach to a simple yet realistic reduction cascade, CO_2_ reduction on a Ag surface, and compare different MHC approaches from another study with BV and MHC rate coefficients from the present study. We find that MHC approaches that do not incorporate information about the zero overpotential regime, for example, by extrapolating from the limiting rate coefficient at high overpotential, may not sufficiently capture the physics of a system and may not be accurate. We also compare the reorganization energies calculated using the present approach with those computed *ab initio* by Gao and co-workers. This work provides independent support for the large reorganization energies calculated, showing that a range of 2–5 eV should be expected for electron-transfer steps of adsorbed species at electrode–electrolyte interfaces, much larger than the typical outer sphere electron-transfer reorganization energy values of 1 eV for solvated redox couples.

We conclude that the present approach establishes simple relationships that can be applied directly to electron-transfer steps and provides a reliable way to estimate the reorganization energies for interfacial reduction reactions that occur on metallic surfaces if some intrinsic system parameters are known or can be calculated. These parameters are the free energy of activation, electronic coupling strength, and density of states, estimated through electronic structure theory methods and/or experimental measurements. Our approach can be used for evaluating the expected magnitudes of rate coefficients, which can be compared to experimental or theoretical estimates available to assess the quality of the corresponding kinetics measurement or calculation. If reorganization energies are available through other sources, using the present MHC approach with them can elucidate the nature of the kinetics of the electron-transfer with regard to mass-transfer limitations or the presence of other factors influencing the local chemical environments.

## Supplementary Material



## Data Availability

The SI.zip file is available from Zenodo at DOI: 10.5281/zenodo.15192244; it contains additional files and data, including spreadsheets, scripts, and plots, used for figures and tables in the main text.

## Appendix: Kinetic Parameters and Units

The parameters and constants used in the BV and MHC equations, along with their respective units, are provided in Table 4.

**Table 4:** Parameters and Constants Used in BV and MHC Equations with Their Respective Units (All Rate Coefficients Are Evaluated in Units of per Second before Necessary Conversions)

Parameter/Constant	Description	Units
Δ*G*_*a*_	activation energy	eV
*α*	symmetry factor	unitless
*η*	overpotential	V
*n*_*e*_	number of electrons	unitless
*ρ*	average density of states	eV^–1^
*H*_*OR*_	electronic coupling strength	eV
*λ*	reorganization energy	eV
*T*	temperature	K
*β*	electronic coupling attenuation coefficient	nm^–1^
*r*_0_, *r*	distances from electrode surface	nm
*k*_∞_	limit of *k*_*red*_^*MHC*^ as *η* → ∞	cm/s
*k*_*B*_	Boltzmann constant	eV/K
*R*	universal gas constant	eV/mol·K
*h*	Planck’s constant	eV·s
*x*	energy of surface electron, variable of integration	eV

## Notes

Notes on calculated density of states, reorganization energy dependence plots, and information about other accompanying files (PDF)
