# Supplementary material for: Approach to Evaluating Reorganization Energies of Interfacial Electrochemical Reactions
Source: ACS Electrochem. 2025 Jul 2;1(9):1766–75. doi: 10.1021/acselectrochem.5c00158 (PMC12415831; doi:10.1021/acselectrochem.5c00158)
Supplement: Supplementary file 1 [file ec5c00158_si_001.pdf]

SUPPORTING INFORMATION

Approach to Evaluating Reorganization Energies of Interfacial Electrochemical Reactions

Karnamohit Ranka[1,2], *Sijia Ke*[1,2,6], *Chenqi Fan*[1,2,4], *Jeffrey B. Neaton*[1,3,5,6,7], *Peter Agbo*[1,2,8], *Frances A. Houle*[1,2,8]*

[1] Liquid Sunlight Alliance, Lawrence Berkeley National Laboratory, Berkeley, California 94720, United States

[2] Chemical Sciences Division, Lawrence Berkeley National Laboratory, Berkeley, California 94720, United States

[3] Department of Physics, University of California at Berkeley, Berkeley, California 94720, United States

[4] Department of Chemistry, University of California at Berkeley, Berkeley, California 94720, United States

[5] Materials Sciences Division, Lawrence Berkeley National Laboratory, Berkeley, California 94720, United States

[6] Department of Materials Science and Engineering, University of California at Berkeley, Berkeley, California 94720, United States

[7] Kavli Energy NanoSciences Institute at Berkeley, Berkeley, California 94720, United States

[8] Molecular Biophysics and Integrated Bioimaging Division, Lawrence Berkeley National Laboratory, Berkeley, California 94720, United States

* Email: fahoule@lbl.gov

Table of contents

Supplementary note SN1. Density of states: computational details

Supplementary note SN2. Surface reactions on Ag: Ag(110) vs Ag(111)

Supplementary note SN3. Notes on supplementary files

A. "plots_data.xlsx"

B. "lambda_minimization.txt"

C. "fig5_plot.py"

Figure S1. CO_2RR on Cu(111): reorganization energy vs activation barriers from Gao *et al.* (PCCP, 2020)

Table S1. Reduction of adsorbates on Ag(111): effect of change in the density of states on reorganization energy trends

Table S2. CO_2RR on Cu(111): present study vs Gao *et al.* (PCCP, 2020)

Table S3. CO_2RR on Cu(111): variation in activation energies, Gao *et al.* (PCCP, 2020) vs Zijlstra *et al.* (Electrochim. Acta, 2020)

Supplementary note SN1. Density of states: computational details

The electrode metal surfaces (Cu(111) and Ag(110)) are modeled with a 13-atom slab and 20 Å vacuum. Our first-principles density functional theory calculations are carried out using the Vienna Ab Initio Simulation Package with projector augmented wave potentials.[1–5] We use the exchange-correlation functional of Perdew, Burke, and Ernzerhof[6] for relaxing the surface and density of states calculations. A Γ-centered $25 \times 25 \times 1$ reciprocal space sampling and a kinetic energy cutoff of 600 eV for the wavefunction are used in self-consistent field calculations, with which the Fermi level and total energy are converged within 3 meV. A Γ-centered $50 \times 50 \times 1$ reciprocal space sampling is further used to obtain the density of states. Experimental lattice parameters for Cu (2.553 Å)[7] and Ag (4.09 Å)[8] are used when constructing surfaces. The density of states is also converged with respect to vacuum size and the number of atoms in slab.

Supplementary note SN2. Surface reactions on Ag: Ag(110) vs Ag(111).

Reaction-specific parameters for aqueous reduction of CO_2 on Ag electrode surface are obtained for the Ag(111) surface. As mentioned in the main article, the electronic coupling strength, H_{OR}, assumed for all adsorbates on the Ag surface is also derived using parameters calculated for the Ag(111) surface structure. However, the density of states (DOS) distribution, ρ, used for calculating the Marcus-Hush-Chidsey rate coefficients in this work, is that for the Ag(110) surface structure. Because the averaged DOS is employed for the calculations, the difference of DOS is not assumed to differ significantly between the two surface structures.[9]

Supplementary note SN3. Notes on supplementary files.

A. "plots_data.xlsx"

Within this file, the sheet titled "l vs DGa (Cu(111))" contains parameters and equations used to obtain the relationship between λ and ΔG_a for a Cu electrode with Cu(111) surface. The sheets titled "l vs HOR (Cu(111))" and "l vs T (Cu(111))" contain relevant data used to obtain relationships between λ and H_{OR}, T, respectively. The sheet titled "l vs DOS (Ag(110))" contains relevant data used to obtain relationships between λ and ΔG_a, H_{OR} for the Ag electrode with Ag(110) surface. The sheet titled "l vs DGa vs HOR (Cu(111))" contains relevant data used to obtain the relationship between λ and ΔG_a, H_{OR} simultaneously, for three different temperatures (298, 350, and 500 K) for the Cu electrode. The sheet titled "CO2RR @ Ag(110)" contains references and relevant data used to calculate the different rate coefficients for CO_2 reduction and hydrogen evolution steps on the Ag electrode with the Ag(110) surface. The E_0 and ΔG_a values for these steps have been obtained from the listed references.[10,11] It additionally contains the data used for making **Figure 1**, the rate coefficient of $CO_2^* \rightarrow COOH^{**}$ as a function of overpotential. The sheet titled "CO2RR @ Cu(111)" contains references and relevant data used to compare and calculate λ as a function of ΔG_a, the E_0 and ΔG_a values having been obtained from the listed reference.[12] The sheets titled "Cu(111)_DOS_surface" and "Ag(110)_DOS_surface" contain the data for the calculated electronic surface density of states as well as the average of these distributions, ρ, according to the procedure outlined in section **Supplementary note SN1**, for the Cu and Ag electrodes.

B. "lambda_minimization.txt"

This file contains the Visual Basic subroutine used for minimization of the difference between the MHC and BV rate coefficients, $|k_{red}^{MHC} - k_{red}^{BV}|$, as a function of λ to obtain the optimized λ, λ_{opt}.

C. "fig5_plot.py"

This file contains the Python program used for plotting **Figure 5**. It utilizes the .CSV files with the prefix "fig5_data_" for data input. The .CSV files with the data themselves are obtained from the sheet titled "l vs DGa vs HOR (Cu(111))" within the file "plots_data.xlsx". The program generates three sub-plots, titled "fig5_plot_298K_YlGn.png", "fig5_plot_350K_YlGn.png" and "fig5_plot_500K_YlGn.png", that can be combined to obtain **Figure 5**.

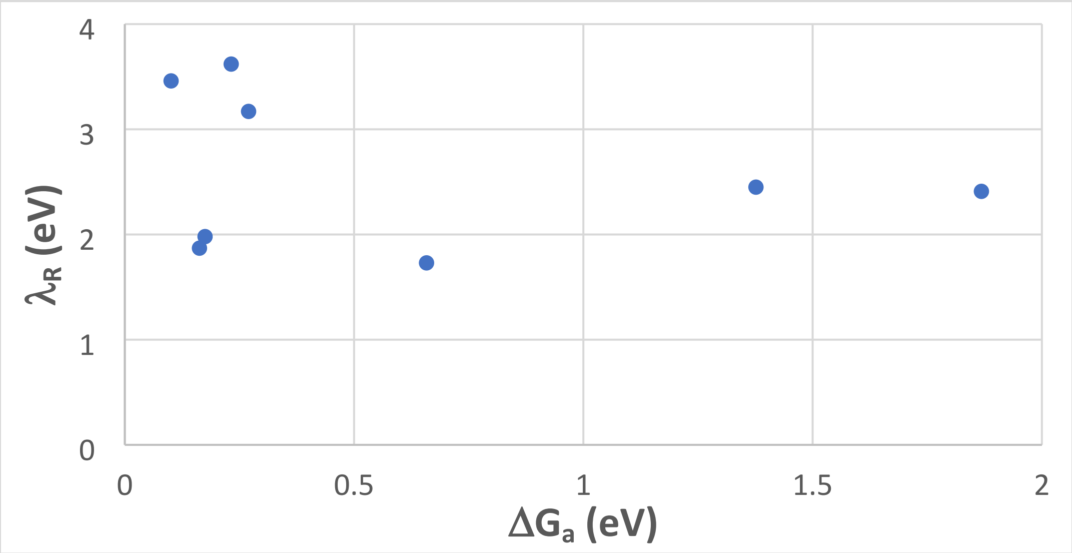

Figure S1. CO_2RR on Cu(111): reorganization energies, λ_R, plotted as a function of ΔG_a, both from Gao *et al.* (PCCP, 2020)[12].

Table S1. Reorganization energy trends for adsorbate reduction reactions on Ag(111) surface, calculated using equation (7).

$\lambda = m\,\Delta G_a + b$	$H_{OR} = 10^{-3}$ **eV**		$H_{OR} = 10^{-6}$ **eV**	
	m	b	m	b
$T = 298$ **K**	1.9969	1.4282	1.9944	0.7232
$T = 1000$ **K**	1.9969	4.5899	–	–
$\lambda = o\ln(H_{OR}) + d$	$T = 298$ **K**			
	o	d		
$\Delta G_a = 0.1$ **eV**	0.102	2.333		
$\Delta G_a = 0.7$ **eV**	0.1024	3.5327		
$\Delta G_a = 1.5$ **eV**	0.1026	5.1319		

Table S2. CO_2RR on Cu(111) surface: comparison of reorganization energies calculated in the present study with those calculated in the study by Gao *et al.* (PCCP, 2020)[12].

**Reaction/TS code (for Cu(111) surface)**[a]	ΔG_a ($\eta = 0$) **(eV)**[a]	E_0 **(V vs SHE)**[a]	a **(/V)**[a]	b[a]	c **(V)**[a]	λ_R **(eV)**[a]	λ_P **(eV)**[a]	λ_{opt} ($T = 298$ **K,** $H_{OR} = 10^{-3}$ **eV) (eV)**[b]	λ_{opt} ($T = 298$ **K,** $H_{OR} = 10^{-6}$ **eV) (eV)**[b]
C1/TS1	1.38	0.6	0.09	0.59	0.99	2.45	2.89	4.20	3.49
C2/TS2	0.10	−0.9	0.09	0.38	0.37	3.46	2.82	1.65	0.94
C3/TS3	1.87	0.9	0.11	0.71	1.14	2.41	2.18	5.18	4.47
C4/TS4	0.66	0.2	0.15	0.56	0.54	1.73	1.71	2.76	2.06
C5/TS5	0.23	-0.6	0.1	0.44	0.46	3.62	2.58	1.91	1.21
C6/TS6	0.18	-0.5	0.1	0.36	0.33	1.98	2.38	1.80	1.09
C7/TS7	0.16	-0.8	0.07	0.29	0.35	1.87	3.40	1.77	1.07
C8/TS8	0.27	-0.84	0.06	0.36	0.53	3.17	3.94	1.99	1.28

[a] from Gao *et al.*, *Phys. Chem. Chem. Phys.*, 22, pp 9607-9615 (2020)

[b] present study

Table S3. CO_2RR on Cu(111) surface: calculated activation barriers from Gao *et al.* (PCCP, 2020)[12] and Zijlstra *et al.* (Electrochim. Acta, 2020)[13].

Reaction/TS code (for Cu(111) surface)[a]	ΔG_a ($\eta = 0$) (eV)[a]	ΔG_a ($\eta = 0$) (eV)[c]	E_0 (V vs SHE)[a]	E_0 (V vs SHE)[c]
C1/TS1	1.38	1.83	0.6	0.717
C2/TS2	0.10	0.43	−0.9	−1.048
C3/TS3	1.87	1.54	0.9	0.997
C4/TS4	0.66	0.92	0.2	0.497

C5/TS5	0.23	0.13	−0.6	−0.863
C6/TS6	0.18	0.30	−0.5	−0.368
C7/TS7	0.16	0.21	−0.8	−0.613
C8/TS8	0.27	0.40	−0.84	−0.678

[a] from Gao *et al.*, *Phys. Chem. Chem. Phys.*, 22, pp 9607-9615 (2020)

[c] from Zijlstra *et al.*, *Electrochim. Acta*, 335, pp 135665 (2020)

REFERENCES

(1) Kresse, G.; Hafner, J. *Ab Initio* Molecular Dynamics for Liquid Metals. *Phys. Rev. B* **1993**, *47* (1), 558–561. https://doi.org/10.1103/PhysRevB.47.558.

(2) Kresse, G.; Hafner, J. *Ab Initio* Molecular-Dynamics Simulation of the Liquid-Metal–Amorphous-Semiconductor Transition in Germanium. *Phys. Rev. B* **1994**, *49* (20), 14251–14269. https://doi.org/10.1103/PhysRevB.49.14251.

(3) Kresse, G.; Furthmüller, J. Efficient Iterative Schemes for *Ab Initio* Total-Energy Calculations Using a Plane-Wave Basis Set. *Phys. Rev. B* **1996**, *54* (16), 11169–11186. https://doi.org/10.1103/PhysRevB.54.11169.

(4) Kresse, G.; Furthmüller, J. Efficiency of Ab-Initio Total Energy Calculations for Metals and Semiconductors Using a Plane-Wave Basis Set. *Comput. Mater. Sci.* **1996**, *6* (1), 15–50. https://doi.org/10.1016/0927-0256(96)00008-0.

(5) Kresse, G.; Joubert, D. From Ultrasoft Pseudopotentials to the Projector Augmented-Wave Method. *Phys. Rev. B* **1999**, *59* (3), 1758–1775. https://doi.org/10.1103/PhysRevB.59.1758.

(6) Perdew, J. P.; Burke, K.; Ernzerhof, M. Generalized Gradient Approximation Made Simple. *Phys. Rev. Lett.* **1996**, *77* (18), 3865–3868. https://doi.org/10.1103/PhysRevLett.77.3865.

(7) Crljen, Ž.; Lazić, P.; Šokčević, D.; Brako, R. Relaxation and Reconstruction on (111) Surfaces of Au, Pt, and Cu. *Phys. Rev. B* **2003**, *68* (19), 195411. https://doi.org/10.1103/PhysRevB.68.195411.

(8) Narasimhan, S. Ab Initio Lattice Dynamics of Ag(110). *Surf. Sci.* **2002**, *496* (3), 331–344. https://doi.org/10.1016/S0039-6028(01)01619-3.

(9) Herrera-Suárez, H. J.; Rubio-Ponce, A.; Olguín, D. Electronic Band Structure of Silver Low-Index Surfaces: A Tight-Binding Study. *Can. J. Phys.* **2020**, *98* (5), 488–496. https://doi.org/10.1139/cjp-2019-0218.

(10) Chen, L. D.; Urushihara, M.; Chan, K.; Nørskov, J. K. Electric Field Effects in Electrochemical CO_2 Reduction. *ACS Catal.* **2016**, *6* (10), 7133–7139. https://doi.org/10.1021/acscatal.6b02299.

(11) Tang, M. T.; Liu, X.; Ji, Y.; Norskov, J. K.; Chan, K. Modeling Hydrogen Evolution Reaction Kinetics through Explicit Water–Metal Interfaces. *J. Phys. Chem. C* **2020**, *124* (51), 28083–28092. https://doi.org/10.1021/acs.jpcc.0c08310.

(12) Gao, S.-T.; Xiang, S.-Q.; Shi, J.-L.; Zhang, W.; Zhao, L.-B. Theoretical Understanding of the Electrochemical Reaction Barrier: A Kinetic Study of CO_2 Reduction Reaction on Copper Electrodes. *Phys. Chem. Chem. Phys.* **2020**, *22* (17), 9607–9615. https://doi.org/10.1039/C9CP06824D.

(13) Zijlstra, B.; Zhang, X.; Liu, J.-X.; Filot, I. A. W.; Zhou, Z.; Sun, S.; Hensen, E. J. M. First-Principles Microkinetics Simulations of Electrochemical Reduction of CO2 over Cu Catalysts. *Electrochimica Acta* **2020**, *335*, 135665. https://doi.org/10.1016/j.electacta.2020.135665.
